# Polyploidy and hybridization in the Mediterranean: unravelling the evolutionary history of *Centaurium* (Gentianaceae)

**DOI:** 10.1093/aob/mcae066

**Published:** 2024-04-30

**Authors:** Ana Valdés-Florido, Claudia González-Toral, Enrique Maguilla, Eduardo Cires, Zoila Díaz-Lifante, Cristina Andrés-Camacho, Gonzalo Nieto Feliner, Juan Arroyo, Marcial Escudero

**Affiliations:** Department of Plant Biology and Ecology, Faculty of Biology, University of Seville, Seville, 41012, Spain; Department of Organisms and Systems Biology, University of Oviedo, Oviedo, 33071, Spain; Department of Molecular Biology and Biochemical Engineering, Pablo de Olavide University, Seville, 41013, Spain; Department of Organisms and Systems Biology, University of Oviedo, Oviedo, 33071, Spain; Institute of Natural Resources and Territorial Planning (INDUROT), Campus de Mieres, Mieres, 33600, Spain; Department of Plant Biology and Ecology, Faculty of Biology, University of Seville, Seville, 41012, Spain; Department of Plant Biology and Ecology, Faculty of Biology, University of Seville, Seville, 41012, Spain; Real Jardín Botánico (RJB), CSIC, Plaza de Murillo 2, Madrid, 28014, Spain; Department of Plant Biology and Ecology, Faculty of Biology, University of Seville, Seville, 41012, Spain; Department of Plant Biology and Ecology, Faculty of Biology, University of Seville, Seville, 41012, Spain

**Keywords:** Allopolyploidy, centauries, hybridization, Mediterranean, plant evolution, polyploidy, RADseq

## Abstract

**Background and Aims:**

Polyploidy is considered one of the main mechanisms of plant evolution and speciation. In the Mediterranean Basin, polyploidy has contributed to making this region a biodiversity hotspot, along with its geological and climatic history and other ecological and biogeographical factors. The Mediterranean genus *Centaurium* (Gentianaceae) comprises ~25 species, of which 60 % are polyploids, including tetraploids and hexaploids. To date, the evolutionary history of centauries has been studied using Sanger sequencing phylogenies, which have been insufficient to fully understand the phylogenetic relationships in this lineage. The goal of this study is to gain a better understanding of the evolutionary history of *Centaurium* by exploring the mechanisms that have driven its diversification, specifically hybridization and polyploidy. We aim to identify the parentage of hybrid species, at the species or clade level, as well as assessing whether morphological traits are associated with particular ploidy levels.

**Methods:**

We sequenced RADseq markers from 42 samples of 28 *Centaurium* taxa, and performed phylogenomic analyses using maximum likelihood, summary coalescent SVDquartets and Neighbor-Net approaches. To identify hybrid taxa, we used PhyloNetworks and the fastSTRUCTURE algorithm. To infer the putative parental species of the allopolyploids, we employed genomic analyses (SNIPloid). The association between different traits and particular ploidy levels was explored with non-metric multidimensional scaling.

**Key Results:**

Our phylogenetic analyses confirmed the long-suspected occurrence of recurrent hybridization. The allopolyploid origin of the tetraploid *C. serpentinicola* and the hexaploids *C. mairei*, *C. malzacianum* and *C. centaurioides* was also confirmed, unlike that of *C. discolor*. We inferred additional signatures of hybridization events within the genus and identified morphological traits differentially distributed in different ploidy levels.

**Conclusions:**

This study highlights the important role that hybridization has played in the evolution of a Mediterranean genus such as *Centaurium*, leading to a polyploid complex, which facilitated its diversification and may exemplify that of other Mediterranean groups.

## INTRODUCTION

Polyploidy, originally defined as the possession of three or more chromosome sets in each cell (Grant, 1981), is considered a key driver of plant evolution and speciation (Stebbins, 1947, 1950; Grant, 1981; Ramsey and Schemske, 1998; Soltis *et al*., 2009). Indeed, almost 35 % of angiosperms have been reported to be recent polyploids (Stebbins, 1971; Grant, 1981; Wood *et al*., 2009). Meanwhile, genomic data have revealed that most flowering plants include in their evolutionary history rounds of polyploidization followed by post-polyploid diploidization (Wendel, 2015; Escudero and Wendel, 2020). Polyploids have been generally classified into two categories: autopolyploids, which form from unreduced gametes of the same species, and allopolyploids, which result from hybridization between different species (Stebbins, 1947; Grant, 1981; Tate *et al*., 2005).

It has been suggested that polyploidy and hybridization have been critical evolutionary processes shaping the evolution and diversification of the Mediterranean flora (Thompson, 2005; Marques *et al*., 2018; Nieto Feliner *et al*., 2023). The geological and climatic history of the Mediterranean area has allowed these evolutionary mechanisms to play an important role, contributing to the establishment of this area as a biodiversity hotspot (Marques *et al*., 2018). Processes such as the Messinian salinity crisis (Duggen *et al*., 2003), the onset of the Mediterranean climate (Suc, 1984) and the climatic changes during the Pleistocene (Hewitt, 2000) provided a suitable arena for these evolutionary mechanisms (Nieto Feliner *et al*., 2023). On one hand, hybridization events could have resulted from the contact between previously isolated lineages when climate regime oscillation processes combined with successive changes in land connections during different geological events led to changes in species’ distribution ranges (Hewitt, 2000; Thompson, 2005; Nieto Feliner, 2014). On the other hand, polyploidization and hybridization have been associated with the emergence of new traits that could facilitate the colonization of new areas or confer different abilities to cope with climate change (Levin, 2002; Marques *et al*., 2016; Vallejo-Marín *et al*., 2016). Finally, an increase in the production of unreduced gametes has been associated with environmental stress from the climatic changes recorded in the Mediterranean (Ramsey and Schemske, 1998; Brownfield and Köhler, 2011; Mason and Pires, 2015). Mediterranean genera such as *Narcissus* (Santos-Gally *et al*., 2012), *Phlomis* (Albaladejo and Aparicio, 2007) and *Centaurium* (Mansion *et al*., 2005; Jiménez-Lobato *et al*., 2019; Maguilla *et al*., 2021) have been proposed to have experienced these processes.

The genus *Centaurium* (Gentianaceae), commonly known as centauries, comprises ~25 species (Mansion and Struwe, 2004; Díaz-Lifante, 2012; Plants of the World Online, 2023). This genus is distributed in temperate and arid climate regions of Asia, Europe, north-central Africa and North America (Supplementary Data Fig. S1), and its centre of diversity is the Mediterranean Basin (Maguilla *et al*., 2021). Zeltner (1970) found two basic chromosome numbers, *x* = 9 and *x* = 10, and three ploidy levels: diploid (2*n* = 2*x* = 18, 20), tetraploid (2*n* = 4*x* = 36, 40) and hexaploid (2*n* = 6*x* = 54, 56, 60) (Table 1). There are a few species that have more than one ploidy ploidy level. For instance, there are diploid and tetraploid individuals in *C. portense* Butcher and *C. serpentinicola* Carlström, and tetraploid and hexaploid individuals in *C. scilloides* (L. fil.) Samp. and *C. turcicum* (Velen.) Ronniger. Two species include three ploidy levels (2*x*, 4*x*, 6*x*): *C. tenuiflorum* (Hoffmanns. & Link) Fritsch and *C. pulchellum* (Sw.) Druce, although there is only one known diploid population of *C. pulchellum*, located in Israel (Zeltner, 1985). Around 60 % of the taxa in *Centaurium* are polyploids (Zeltner, 1970; Mansion *et al*., 2005), which suggests that polyploidy has been a significant force in the evolutionary history of this genus. Interestingly, ploidy levels seem to follow a geographical pattern. Diploids (2*x*) are mainly distributed in the Mediterranean Basin, tetraploids (4*x*) in Northern Europe and Eastern Asia, and hexaploids (6*x*) in the south-western Mediterranean Basin and on the Arabian Peninsula (Mansion *et al*., 2005; Prieto *et al*., 2012; Maguilla *et al*., 2021). At a finer scale within the Mediterranean Basin, ploidy levels are also not randomly distributed, with tetraploids dominating at upper latitudes, hexaploids more common at lower latitudes, and diploids inhabiting the core of the Mediterranean Basin (Mansion *et al*., 2005). Thus, the biogeographical study of *Centaurium* supports the hypothesis that polyploidy played an important role in the spread of the genus, suggesting that ancestral diploid species remained in the likely area of origin (i.e. the Mediterranean Basin), whereas polyploids expanded into new areas (Maguilla *et al*., 2021). Both allopolyploidy and autopolyploidy have been reported among *Centaurium* species using karyological and phylogenetic evidence, and it has been tentatively suggested that the hexaploids are allopolyploids while tetraploids are autopolyploids (Zeltner, 1970; Mansion *et al*., 2005). However, inferring the auto- or allopolyploid origin of a polyploid species is challenging, because of the intermediate levels of differentiation in genomes from conspecific populations and the dynamism of merged genomes following whole-genome duplication. In this genus, only one allopolyploid origin is documented with enough certainty: Guggisberg *et al*. (2006) reported *C. discolor* (Gand.) Ronniger as an allotetraploid derived from *C. maritimum* (L.) Fritsch and *C. tenuiflorum* using molecular analyses (RAPD analyses) and flow cytometry.

**Table 1.** Studied *Centaurium* taxa, locality, collector, voucher, collection date, internal code, ploidy level, source of the sample (i.e. herbarium material or silica-gel dried) and code used for PhyloNetworks analysis.

Species	Locality	Collector	Voucher	Collection date	Code	Ploidy level	Source	Code for PhyloNetworks analyses
*C. capense* Broome	Mexico: Baja California, Bocana Rio	L. & N. Zeltner	NEU-2605	06/06/1998	CENT-53	4*x*	Herbarium material	Cap
*C. centaurioides* R.S. Rao & Hemadri	India: Maharashtra, Ramtek	L. & N. Zeltner	NEU-1674	25/03/1991	CENT-51	6*x*	Herbarium material	Cent
*C. chloodes* (Brot.) Samp.	Spain: Coruña, Muxia	Louzán & Casais	SANT-45049	23/05/2001	CENT-26	4*x*	Herbarium material	Port
*C. discolor* (Gand.) Ronniger	Spain: Mallorca, Algaida	C. Navarro & al.	MA-618284	02/06/1998	CENT-25	4*x*	Herbarium material	Dis
*C. erythraea* Rafn subsp. *erythraea*	France: Cevennes, Vebrori	J. Arroyo	SEV-270216	06/06/2015	CENT-64	4*x*	Herbarium material	Ery2
*C. erythraea* Rafn subsp. *erythraea*	Spain: Los Molinos, Sierra de Guara	J. Viruel	SEV-241216	23/06/2009	CENT-3	4*x*	Herbarium material	Ery2
*C. erythraea* subsp*. rhodense* (Boiss. & Reut.) Melderis	Italy: Sicilia, San Fratello	L. & N. Zeltner	NEU-3517	19/06/2002	CENT-77	4x	Herbarium material	Ery2
*C. erythraea* subsp*. rhodense* (Boiss. & Reut.) Melderis	Sicily: Portella	Z. Díaz	–	04/06/2017	CAV-2.2	4*x*	Silica-gel dried	Ery2
*C. erythraea* subsp*. rumelicum* (Velen.) Melderis	Bulgary: Fargovo	J. Aldasoro	VAL-163793	06/07/2004	CENT-36	2*x*	Herbarium material	Maj
*C. erythraea* var*. subcapitatum* (Corb.) Ubsdell	Ireland: Galway, Carraroe, Connemara	L. & N. Zeltner	NEU-2869	04/08/1996	CENT-54	4*x*	Herbarium material	Ery2
*C. grandiflorum* subsp*. boissieri* (Willk.) Z. Díaz	Spain: Málaga, Ardales	C. Andrés & Z. Diaz	SEV-299926	23/06/2008	CENT-4	2*x*	Herbarium material	Boi
*C. grandiflorum* subsp*. boissieri* (Willk.) Z. Díaz	Spain: Sevilla, Universidad Pablo de Olavide	Z. Díaz	-	16/04/2015	CAV-5	2*x*	Silica-gel dried	Boi
*C. grandiflorum* (Pers.) Ronniger subsp. *grandiflorum*	Spain: Castellón, Morella	Z. Díaz	SEV-241188	02/07/2009	CENT-6	2x	Herbarium material	Grand
*C. grandiflorum* (Pers.) Ronniger subsp. *grandiflorum*	France: Corbières, Les Sauzils	J. Arroyo	SEV-270215	02/07/2015	CENT-68	2*x*	Herbarium material	Grand
*C. grandiflorum* subsp. *majus* (Hoffmanns. & Link) Z. Díaz	Italy: Cerdeña, Nuoro	M.A. García & al.	VAL-173304	06/06/2003	CENT-31	2*x*	Herbarium material	Maj
*C. grandiflorum* subsp. *majus* (Hoffmanns. & Link) Z. Díaz	Spain: Huelva, Aroche	F. Pina	SEV-224140	15/05/2007	CENT-9	2*x*	Herbarium material	Maj
*C. littorale* (Turner) Gilmour subsp. *littorale*	Denmark: Jutland, Faerfer Vig, Fur, Ile	L. & N. Zeltner	NEU-2980	14/07/2001	CENT-55	4*x*	Herbarium material	Lit2
*C. littorale* (Turner) Gilmour subsp. *littorale*	Denmark: Jutland, Skallingen D27	L. & N. Zeltner	NEU-2988	16/06/2001	CENT-76	4*x*	Herbarium material	Lit2
*C. littorale* subsp. *uliginosum* (Waldst. & Kit.) Melderis	Denmark: Jutland, Skallingen D27	L. & N. Zeltner	NEU-2989	16/07/2001	CENT-56	4*x*	Herbarium material	Lit2
*C. littorale* subsp. *uliginosum* (Waldst. & Kit.) Melderis	Sweden: Gotland, Herrvik prope Katthammarsvik	L. & N. Zeltner	NEU-1699	06/08/1990	CENT-71	4*x*	Herbarium material	Ulig1
*C. mairei* Zeltner	Niger: Aïr, Monts des Bagzanes	L. & N. Zeltner	NEU-1740	28/03/1986	CENT-70	6*x*	Herbarium material	Cap
*C. malzacianum* Maire	Oman: Al Akhdar, At tayyib, Wadi Beni Ghafir	G. Mansion & N. Zeltner	NEU-2844	04/04/2000	CENT-72	6*x*	Herbarium material	Mal
*C. maritimum* (L.) Fritsch	Portugal: Cruz de João Mendes	Z. Díaz & V. Girón	SEV-224120	30/04/2008	CENT-29	2*x*	Herbarium material	Mar
*C. maritimum* (L.) Fritsch	Spain: Cádiz, Algeciras, Zanona	Z. Díaz	MAR-551	22/05/2018	CENT-29.2	2*x*	Silica-gel dried	Mar
*C. portense* Butcher	Spain: Piélagos, Cantabria	Ceballos & Fdez. Prieto	FCO-32680	10/09/2011	CPO-1.2	2*x*	Silica-gel dried	Port
*C. pulchellum* (Sw.) Druce	Croatia: Trogir island	J. Arroyo & A. Castro	SEV-270218	19/06/2015	CENT-13	4*x*	Herbarium material	Pul
*C. pulchellum* (Sw.) Druce	Portugal: Aveiro, Puente de Barra	Z. Díaz & A. Castro	SEV-270223	07/05/2015	CENT-22	4*x*	Herbarium material	Pul
*C. quadrifolium* subsp. *barrelieri* (L.M. Dufour) G. López	Spain: Valencia, Els Corrals	Z. Díaz	SEV-241173	01/07/2009	CENT-14	2*x*	Herbarium material	Qua
*C. quadrifolium* subsp. *barrelieri* (L.M. Dufour) G. López	Spain: Valencia, Barx	Z. Díaz	–	21/06/2019	CAV-11.2	2*x*	Silica-gel dried	Qua
*C. quadrifolium* subsp*. linariifolium* (Lam.) G. López	Spain: Castellón, Mosqueruela	Z. Díaz	SEV-241185	02/07/2009	CENT-15	2*x*	Herbarium material	Lin
*C. quadrifolium* subsp*. linariifolium* (Lam.) G. López	Spain: Granada, Frigiliana	B. Cabezudo & al.	MGC-57199	23/05/2003	CENT-32	2*x*	Herbarium material	Lin
*C. quadrifolium* subsp*. parviflorum* (Willk.) Pedrol	Spain: Segovia, Hinojosa del Cerro	Z. Díaz	SEV-270224	21/07/2015	CENT-60	2*x*	Herbarium material	Qua
*C. quadrifolium* subsp*. quadrifolium* (L.) G. López & C.E. Jarvis	Spain: Madrid, Villaconejos-Chinchón	Z. Díaz & F. García	–	23/07/2015	CAV-15	2*x*	Silica-gel dried	Qua
*C. quadrifolium* subsp*. quadrifolium* (L.) G. López & C.E. Jarvis	Spain: Toledo	Z. Díaz	–	19/07/2016	12 quadri (TO)	2*x*	Silica-gel dried	Qua
*C. scilloides* (L. fil.) Samp.	Spain: Coruña, Porto do Son	R. Carballal	SANT-57987	20/10/2008	CENT-37	2*x*	Herbarium material	Port
*C. scilloides* (L. fil.) Samp.	Azores, Ilha do Pico	Bueno & Fdez. Prieto	FCO-32672	16/06/2011	CSC-1 (CAV-27)	2*x*	Silica-gel dried	Port
*C. serpentinicola* Carlström	Turkey: Mugla, Carie, Köycegiz	L. & N. Zeltner	NEU-2998	07/07/2000	CENT-74	4*x*	Herbarium material	Serp
*C. somedanum* M. Laínz	Spain: Oviedo, Pola de Somiedo	Z. Díaz	SEV-270225	21/07/2015	CENT-59	4*x*	Herbarium material	Port
*C. somedanum* M. Laínz	Spain: Oviedo, Valle de Lago	C. Andrés	SEV-270226	20/08/2015	CENT-63	4*x*	Herbarium material	Ulig1
*C. suffruticosum* (Griseb.) Ronniger	Spain: Cádiz, Vejer	V. Girón	SEV-224201	01/05/2009	CENT-16	2*x*	Herbarium material	Maj
*C. tenuiflorum* (Hoffmanns. & Link) Fritsch	Croatia: Peljesac Peninsula, Zabrde	J. Arroyo & A. Castro	SEV-270219	19/06/2015	CENT-18	2*x*	Herbarium material	Ten
*C. turcicum* (Velen.) Ronniger	Turkey: Sinop	Valcárcel & al.	VAL-146633	22/06/2001	CENT-28	4*x*	Herbarium material	Ery2
*Blackstonia perfoliata*	Spain: Huesca, Los Molinos, Sierra de Guara	J. Viruel	SEV 241216	23/06/2009	CENT-19	–	Herbarium material	Out
*Exaculum pusillum*	Spain: Huelva, Embalse Grande	Z. Díaz & V. Girón	SEV-270228	03/06/2010	CENT-24	–	Herbarium material	Out
*Schenkia spicata*	Spain: Albacete, Cordovilla	Z. Díaz	SEV-270220	11/08/2010	CENT-20	–	Herbarium material	Out

Several phylogenetic studies have reconstructed the evolutionary history of the genus using Sanger sequencing data from several nuclear and plastid regions. Mansion and Struwe (2004) and Mansion *et al*. (2005) used the nuclear internal transcribed spacer (*ITS*) and the plastid regions from the *trnL* intron and *trnL-F* spacer. More recently, Jiménez-Lobato *et al*. (2019) used two other nuclear DNA regions [external transcribed spacer (*ETS*) and *NADPH-*cytochrome P450 reductase, *CPR1*] in addition to the *ITS*, as well as several plastid regions: *trnL*, *matK*, *rpoB*, *rpoC*, *trnL-F*, *psbA-trnH* and *atpF-atpH*. Both nuclear and plastid reconstructions supported the monophyly of *Centaurium*. The topologies obtained in the phylogenetic and biogeographic studies by Jiménez-Lobato *et al*. (2019) and Maguilla *et al*. (2021) are consistent with those obtained by Mansion *et al*. (2005) in reconstructing two main clades: the ‘western’ clade, which includes most of the western Mediterranean species, and the ‘widespread’ clade, composed of the more widely distributed species.

To study polyploidy in the genus, Mansion *et al*. (2005) performed phylogenetic analyses on datasets containing only diploid species and on datasets containing both diploid and polyploid species. With the diploid dataset there was no incongruence between nuclear and plastid DNA trees. However, the incongruence length difference (ILD) test found a lack of congruence between plastid and nuclear trees when diploid and polyploid species were analysed together. The authors concluded that intensive reticulation plus polyploidy was the most reasonable explanation for their results, supporting the idea that polyploidy and hybridization play an essential role in the evolutionary history of the genus (Mansion *et al*., 2005).

Although these studies have shed light on the relationships among species, the phylogenetic relationships at shallow nodes remained poorly resolved (Mansion and Struwe, 2004; Mansion *et al*., 2005; Prieto *et al*., 2012; Jiménez-Lobato *et al*., 2019; Maguilla *et al*., 2021). This highlights the need for genomic approaches to better understand the evolutionary history of *Centaurium*, especially if complex mechanisms such as polyploidy and hybridization have contributed to shaping its current diversity.

Restriction site-associated DNA sequencing (RADseq) allows the sequencing of millions of single-nucleotide polymorphisms (SNPs) using restriction enzymes. The large number of SNPs have proven useful for reconstructing the evolutionary history and polyploid origin of species in phylogenetic frameworks, including non-model organisms (Dufresne *et al*., 2014). However, conflicts among gene trees are common in groups that have suffered from processes such as incomplete lineage sorting, hybridization and/or introgression (Maddison, 1997). The study of the relationships among polyploid taxa – especially allopolyploids – has been limited because the subgenomes convey different phylogenetic signals. In these cases, using only classical bifurcating phylogenetic trees is not the best approach to unravel their evolutionary history, and network approaches may be more informative. Polyploid genera such as *Salix*, *Fothergilla* and *Dactylorhiza* (Qi *et al*., 2015; Brandrud *et al*., 2020; He *et al*., 2020; Wagner *et al*., 2020) have been insightfully studied using RADseq approaches.

Differences in morphological traits between diploids and polyploids have been documented (Stebbins, 1947, 1950; Husband and Schemske, 2000). For example, several studies indicate a positive correlation between genome size and cell size (Müntzing, 1936; Otto and Whitton, 2000; Gregory, 2001; Beaulieu *et al*., 2008), so that as ploidy level increases there is a corresponding increase in the size of cells and structures. Specifically, it has been proposed that polyploids exhibit larger leaves and flowers compared with diploids (e.g. Husband and Schemske, 2000; Li *et al*., 2010; Balao *et al*., 2011; Kim *et al*., 2012; Laport and Ramsey, 2015; Etterson *et al*., 2016). However, important reproductive traits (floral size and herkogamy) have been failed to be associated with ploidy levels in genus *Centaurium* (Jiménez-Lobato *et al*., 2019).

In this context, we use RADseq markers to (1) reconstruct the phylogenetic relationships among *Centaurium* species and subspecies, (2) infer the incidence of hybridization in the genus by identifying hybrid species and their parentage at the species or lineage level, and (3) explore morphological traits that may be associated with specific ploidy levels. In doing so, we aim to provide further insight into specific case studies that may contribute to estimating the evolution of polyploidy and hybridization in the Mediterranean flora.

## MATERIALS AND METHODS

### Plant material

A total of 42 samples belonging to 28 taxa of *Centaurium* (15 species and 13 subspecies) encompassing the whole range of distribution were included in this study (Table 1). *Exaculum pusillum* Caruel, *Schenkia spicata* (L.) G. Mans. and *Blackstonia perfoliata* (L.) Huds. were included as outgroups (Table 1). We collected samples from the field and from different herbaria: University of Seville (SEV), University of Oviedo (FCO), Royal Botanic Gardens, Madrid, CSIC (MA), University of Santiago de Compostela (SANT), University of Valencia (VAL), University of Málaga (MGC) and University of Neuchâtel (NEU). We followed the taxonomic treatment proposed by Mansion *et al.*, (2005) and reviewed by Díaz-Lifante (2012). Several *Centaurium* hybrids have received a formal name (*Centaurium* × *aschersonianum* (Seemen) Hegi, *Centaurium* × *cicekii* Yıld. & Yaprak, *Centaurium* × *jolivetinum* P. Fourn. and *Centaurium* × *litardierei* Ronniger, among others). However, these taxa represent occasional hybrids and have not been included in this study, as it focuses on hybridization events that have evolutionary implications.

### DNA extraction, sequencing, and data treatment

DNA extraction was carried out using the DNeasy Plant Mini Kit (Qiagen, Valencia, CA, USA), following the manufacturer’s instructions. DNA concentration was evaluated with a Qubit 2.0 Fluorometer (Life Technologies, Grand Island, NY, USA) and its quality checked on a 1 % agarose gel. RADseq libraries and barcoding were prepared following Baird *et al*. (2008), using the restriction enzyme *Pstl*, by Floragenex Inc. (Beaverton, OR, USA), sequenced on the Illumina HiSeq 2000 platform. Once we had the raw data, we used ipyrad 0.9.65 (Eaton and Overcast, 2020) under a *de novo* assembly for demultiplexing and clustering. The filtering step was performed with a maximum of four low-quality base calls per read, and the minimum length of reads after the adapter trim was set at 35 bp. For the alignment, we used a clustering threshold of 90 %, we set a minimum number of samples per locus of 20, and we allowed at most two alleles per site in consensus sequences.

### Phylogenetic analyses

Phylogenetic relationships were inferred using a maximum likelihood (ML) approach with IQ-TREE 1.6.11 (Minh *et al*., 2020) for all ploidy levels within *Centaurium* (diploid, tetraploid and hexaploid). IQ-TREE analyses were run with the GTR + I + G substitution model (Swofford *et al*., 1996). Statistical support was estimated by 1000 ultrafast bootstrap (UFBoot) replicates (Hoang *et al*., 2018) and by 1000 SH-like approximate likelihood ratio test (SH-aLRT) replicates (Guindon *et al*., 2010). We used the quartet-based method of quartet sampling (QS) to examine the potential phylogenetic discordances of internal and terminal branches with the obtained IQ-TREE topology (Pease *et al*., 2018). We estimated the quartet concordance score (QC), the quartet differential score (QD) and the quartet informativeness score (QI) for each internal node and the quartet fidelity score (QF) for each terminal node by conducting an analysis consisting of four parallel threads and 10 000 replicates. The QC measures the concordance between the QS-derived topology and the test topology (here, the IQ-TREE topology), returning positive values if a given branch is concordant between the two topologies and a negative value if they are discordant. The QD estimates whether the frequencies of two alternative topologies of a discordant branch are similar or favour one over the other; when QD values are close to 1, none of the alternative topologies is favoured, while values close to 0 mean that one of the alternatives is favoured (Pease *et al*., 2018). The QI shows the proportion of informativeness of each replicate; when its value is close to 1, the replicates are informative and when close to 0, the informative replicates descend. QS values (QC/QD/QI) are shown with bootstrap support (BS) values along the phylogeny.

We used the obtained IQ-TREE topology and 10 000 randomly chosen SNPs to obtain 3060 quartets, using four threads and 1000 bootstrap repetitions. As tree topologies cannot completely describe complex evolutionary scenarios such as hybridization (Huson and Bryant, 2006), we performed a Neighbor-Net analysis in SplitsTree4 v. 4.18.3 (Huson and Bryant, 2006) including all ploidy levels within the genus. The genetic distance was inferred by the Uncorrected P method, while statistical support for branches was estimated by 10 000 BS replicates. The recovered network was built by compatible partitions of sets of taxa (or splits; Bryant and Moulton, 2004). The split weight (i.e. branch length) indicates the depth of divergence between taxa, with shorter branches corresponding to genetically similar taxa and longer branches corresponding to more genetically distinct taxa.

Since our dataset included *Centaurium* species with different ploidy levels (2*x*, 4*x*, 6*x*) and they are susceptible to having experienced incomplete lineage sorting, we also used the summary coalescent approach SVDquartets (Chifman and Kubatko, 2015) as implemented in PAUP* 4.0b10 (Swofford, 2002). Thus, a consensus species tree was inferred by analysing 1 000 000 randomly chosen quartets. Branch support was estimated by 10 000 BS repetitions.

### Exploring genetic diversity: identification of hybridization events

Potential hybridization events between lineages occurring in different nodes from the IQ-TREE topology were identified using the SNaQ function of the PhyloNetworks Julia package (Solís-Lemus and Ané, 2016; Solís-Lemus *et al*., 2017). The maximum pseudolikelihood-based SNaQ approach (Solís-Lemus and Ané, 2016) simultaneously infers the main topology (i.e. species relationships) and the underlying historical reticulations (i.e. ancient hybridizations events). We estimated the observed quartet concordance factors (CFs) of our initial alignment in the R package SNPs2CF (Olave and Meyer, 2020), setting the maximum number of SNPs to be used to 15 000 and the maximum number of quartets to 1 000 000, and performing bootstrap replicates to obtain the lower and upper limit of the credibility intervals. For the analyses we used an Imap file (the code used in this analysis is shown in Table 1) considering the presence of multiple individuals per species and, in some clades, several species per clade. This allowed us to establish the individual–clade association. The SNaQ analyses were based on the IQ-TREE topology and the CFs of 3060 quartets. This method consisted of inferring 10 independent topologies, each one repeated 10 times, with different hmax values (i.e. number of different hybridization events). Hmax values ranged from 0 (no hybridization events) to 9. The hmax value was considered the most adequate when the addition of another event did not improve the likelihood score (loglik function). The bootstrap analyses were performed with 100 replicates and 5 runs per replicate.

Our *Centaurium* taxa included three ploidy levels (2*x*, 4*x* and 6*x*), which could include recent and ancient hybridizations or autopolyploid events. Therefore, we aimed to determine which type of polyploidization events (autopolyploid vs allopolyploid) explained both the variety of ploidy levels and the observed phylogenetic relationships. We explored the genetic structure of the *Centaurium* data using Bayesian clustering analysis with fastSTRUCTURE (Raj *et al*., 2014), an algorithm designed to infer population structure from large SNPs in a Bayesian framework. The study was performed for all studied taxa, for *K* values (i.e. the number of genotypic groups) from 2 to 30. We also determined the optimal partition of the data (the *K* value that best explains its genetic structure) with the tool chooseK implemented in fastSTRUCTURE (Raj *et al*., 2014).

We used SNIPloid, developed by Peralta *et al*. (2013), to analyse and classify the SNPs of allopolyploid species to trace them with their putative parentals. This tool was originally designed to classify RNA-Seq SNPs, although Wagner *et al*. (2020) developed a pipeline for RADseq data, by using biallelic SNPs instead of sequence data. This software classifies the SNPs into different categories: categories 1 and 2 correspond to interspecific SNPs that match one of the parental genomes (to parental1 or parental2, respectively) (e.g. parental1 A/A, parental2 G/G, and hybrid G/G). Category 3or4 corresponds to SNPs that do not match either of the diploid parental genomes, as mutations may have occurred in one of the subgenomes of the allopolyploid after the polyploidization event (e.g. parental1 A/A, parental2 A/A, and hybrid A/G). Category 5 comprises the putative homoeo-SNPs (i.e. polymorphisms that occurred in the hybrid species and in the parental genomes) (e.g. parental1 A/A, parental2 G/G, and hybrid A/G). Any SNPs that do not fall into any of the previous categories are classified as other (Peralta *et al*., 2013).

We studied here the allopolyploid origin and parentage of five polyploid species: *C. discolor*, *C. serpentinicola*, *C. malzacianum* Maire, *C. mairei* Zeltner and *C. centaurioides* R.S. Rao & Hemadri. Putative parental species of *C. discolor* (*C. maritimum* and *C. tenuiflorum*) were suggested by Guggisberg *et al*. (2006). To perform our analyses, we used the hypothetical parents proposed by Mansion *et al*. (2005) for the other four polyploids. However, for *C. malzacianum, C. centaurioides* and *C. mairei* we also used partially different parentage suggested by our own previous results from SNIPloid or fastSTRUCTURE. Specifically, we compared the SNPs of *C. serpentinicola* with those of their putative parentals suggested by Mansion *et al*. (2005), i.e. *C. erythraea* subsp. *rumelicum* (Velen.) Melderis and *C. tenuiflorum*. For the hexaploid *C. malzacianum* we considered *C*. *maritimum* and *C. pulchellum*, both from the widespread clade, as putative parentals according to Mansion *et al*. (2005). Additionally, we also used *C. pulchellum* and *C*. *grandiflorum* subsp. *boissieri* (Willk.) Z. Díaz (from the western clade) as potential parental taxa. This followed our previous SNIPloid analysis including *C. maritimum* and *C. pulchellum*, which suggested that *C. pulchellum* and one species from the western clade, not *C. maritimum*, could be the parental species. *Centaurium grandiflorum* subsp. *boissieri* was randomly selected since the parental species from the western clade was not identified. Regarding the hexaploid *C. centaurioides*, we considered *C. pulchellum* and *C. tenuiflorum* as putative parental species according to Mansion *et al*. (2005) in addition to an alternative hypothetical parentage based on the results of our previous SNIPloid analyses consisting of *C. tenuiflorum* and *C. erythraea* Rafn subsp. *erythraea*. SNIPloid suggested that *C. tenuiflorum*, not *C. pulchellum*, and another species from the western clade (*C. erythraea* subsp. *erythraea* was randomly selected) could be the parental taxa. Regarding C. *mairei*, Mansion *et al.* (2005) proposed this taxa to be an autopolyploid from tetraploid populations of *C. pulchellum*. In view of our genetic results, this hexaploid could not be an autopolyploid, as the parental species are included in different genetic clusters. Thus, another species from the western clade was randomly selected: *C. erythraea* subsp. *erythraea*.

### Linking morphology and ploidy level

To explore phenotypic consequences of whole-genome duplication in the evolution of *Centaurium*, we examined whether there is an association between ploidy level and several morphological traits. These were taken from Jiménez-Lobato *et al*. (2019) and consisted of eight morphological traits all coded as binary: flower display, flower size, anther length, androecium symmetry, style position, herkogamy and stigma length or life cycle (annual/biennial vs perennial). We used non-metric multidimensional scaling (NMDS; Kruskal, 1964) to ordinate samples based on these traits (Supplementary Data Table S1). First, we calculated distances between variables under the Jaccard distance method for binary values with the R package vegan, and then we performed the NMDS analysis with the metaMDS function in RStudio (v. 2021.09.2).

## RESULTS

### DNA sequencing

The average number of raw reads from the sequencing was 3.8 million reads per sample. After optimization, we recovered a total of 215 894 filtered RADseq loci. The recovered aligned sequence matrix with 45 accessions had a length of 1 011 716 bp and the SNP matrix a length of 139 464 bp, with 57 437 parsimony-informative sites.

### Phylogenetic inference

**Fig. 1.**
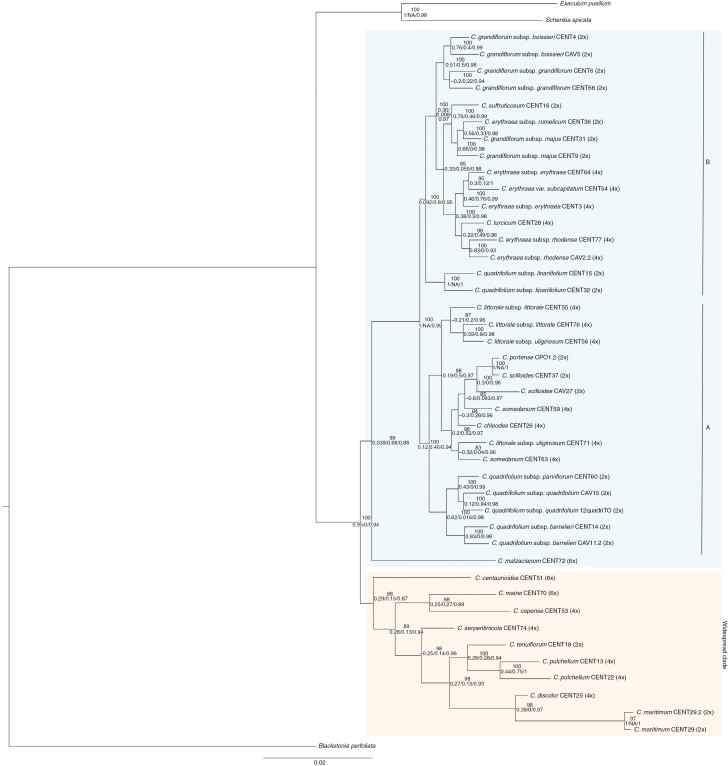
Maximum likelihood phylogenetic reconstruction of all the samples for the genus *Centaurium* performed with RAD markers. Bootstrap and quartet sampling support are displayed at the nodes: bootstrap support, quartet concordance/quartet differential score/quartet informativeness.

The topology recovered from IQ-TREE confirmed the sister relationship of *Exaculum* and *Schenkia* (BS 100, 1/NA/0.98) (Fig. 1). Two main well-supported clades were found within the ingroup: the ‘western’ and the ‘widespread’ clades (Fig. 1), as previously identified in Maguilla *et al*. (2021). The widespread clade (BS 98, 0.29/0.15/0.87) comprises eight species: the hexaploids *C. centaurioides* and *C. mairei* Zeltner, the tetraploids *C. capense* Broome, *C. serpentinicola*, *C. pulchellum* and *C*. *discolor*; and the diploids *C. maritimum* and *C. tenuiflorum.* Among the western clade (BS 99, 0.039/0.68/0.88), two subclades can be recognized, subclades A and B, as well as an accession of the hexaploid *C. malzacianum*. Subclade A (BS 100, 0.12/0.46/0.94) is composed of tetraploid subspecies of *C. littorale* (Turner) Gilmour (subsp. *littorale* and *C. littorale* subsp. *uliginosum* (Waldst. & Kit.) Melderis), the diploids *C. portense* and *C. scilloides*, the tetraploids *C. somedanum* M. Laínz and *C. chloodes* (Brot.) Samp. and, finally, the diploid subspecies of *C. quadrifolium* (subsp. *parviflorum* (Willk.) Pedrol, subsp. *quadrifolium* (L.) G. López & C.E. Jarvis and subsp. *barrelieri* (L.M. Dufour) G. López). Subclade B (BS 100, 0.092/0.8/0.95) is composed of the diploid subspecies of *C. grandiflorum* (subsp. *boissieri*, subsp. *grandiflorum* (Pers.) Ronniger and subsp. *majus* (Hoffmanns. & Link) Z. Díaz), the diploid *C. erythraea* subsp. *rumelicum*, the tetraploids *C. erythraea* subsp. *erythraea*, *C. erythraea* subsp. *rhodense* (Boiss. & Reut.) Melderis and *C. erythraea* var. *subcapitatum* (Corb.) Ubsdell, as well as the tetraploid *C. turcicum* and the diploid *C. suffruticosum* (Griseb.) Ronniger and two accessions of the diploid *C. quadrifolium* subsp. *linariifolium*. (Lam.) G. López.

**Fig. 2.**
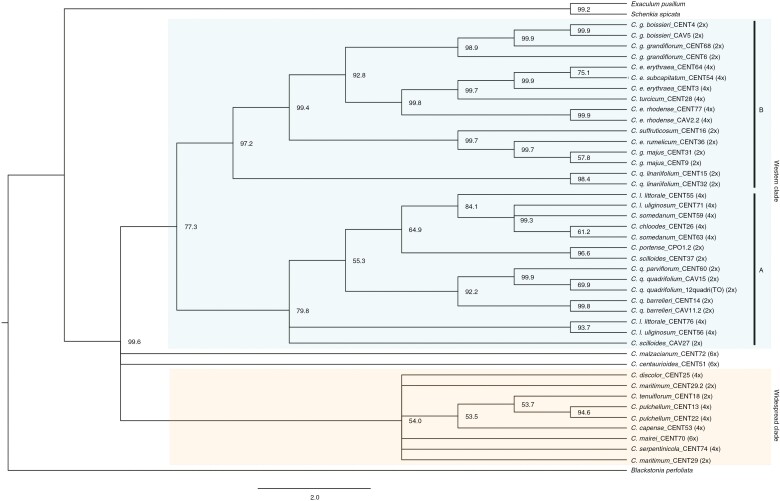
Consensus species tree inferred under the coalescent approach SVDquartets. Taxa, internal code and ploidy level are shown at the tips of the tree. Bootstrap support is shown at the nodes.

Similar to the phylogenetic reconstructions (Fig. 1), the quartet-based tree identifies two main clades within the genus in which most of the nodes had significant support (Fig. 2). However, some polytomies are observed across the tree: one within the western clade affecting the *C. somedanum* CENT59 accession; two involving hexaploid accessions with a haploid number of 28 (*C. malzacianum* and *C. centaurioides*), which were not located in any of the main clades; four within the widespread clade affecting the polyploids *C. discolor* and *C. serpentinicola*, the putative parental species *C. maritimum*, and the hexaploid *C. mairei* accessions. Furthermore, the widespread clade, which had the most polytomies, had no significant support. The polytomies affecting hexaploids and tetraploids may be caused by hybridization events, and in this case the quartet-based method is unable to conclusively resolve the evolutionary relationships among these taxa. The polytomies affecting the accessions of *C. malzacianum* and *C. somedanum* (CENT59) are not unexpected. The first one is one of the main incongruences of our phylogenetic tree and the previous reconstructions of the genus (Jiménez-Lobato *et al*., 2019; see Discussion section), and the latter is reconstructed as paraphyletic in the IQ-TREE reconstruction (Fig. 1).

**Fig. 3.**
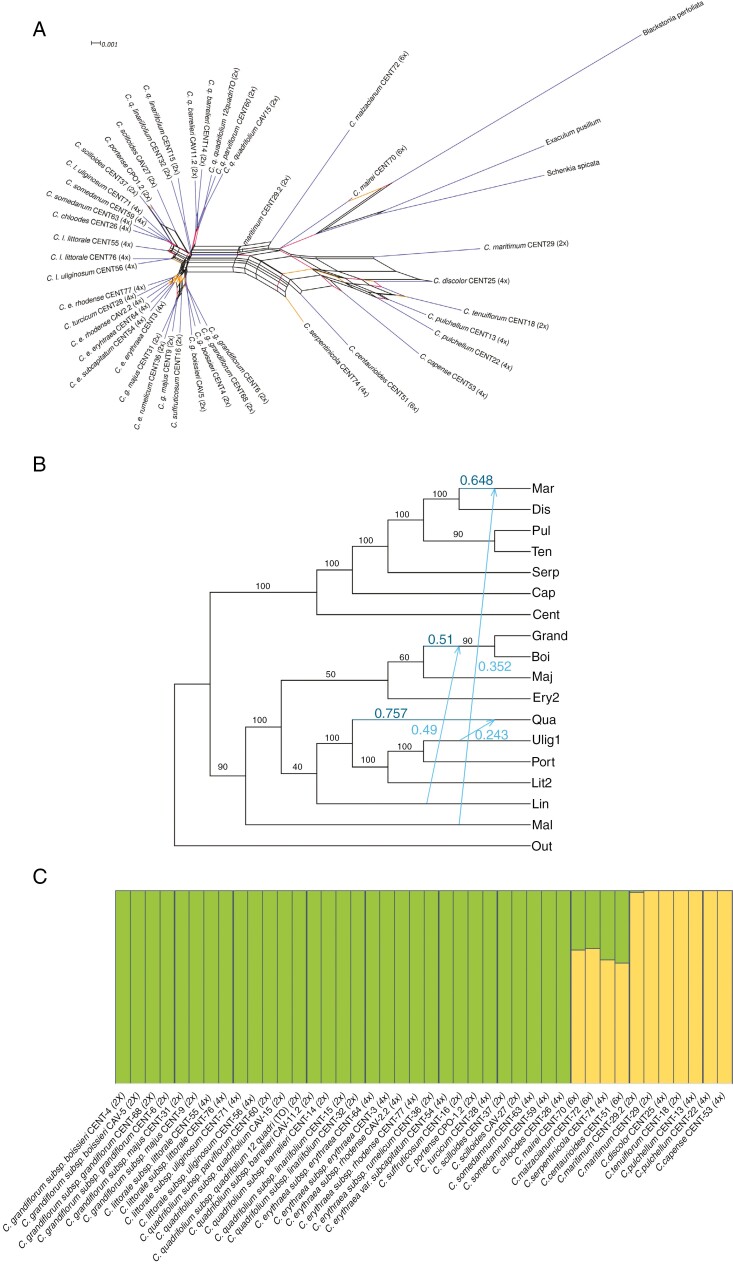
(A) SplitsTree network for diploid, tetraploid and hexaploid species of the genus. Colours indicate bootstrap support: branches with bootstrap support of 0–60 are in red; 60–80 are in orange and 80–100 are in blue. (B) Rooted phylogenetic network with three hybridization events. Inheritance probabilities represent the proportion of genes contributed by each clade/taxa, shown by arrows. The associations of species–individuals are indicated at the tips: Mar corresponds to both accessions of *C. maritimum*, Dis *to C. discolor*, Pul to both accessions of *C. pulchellum*, Ten to *C. tenuiflorum*, Serp to *C. serpentinicola*, Cap to *C. capense* and *C. mairei*, Cent correspond to *C. centaurioides*, Mal to *C. malzacianum*, Qua correspond to both accessions of *C. quadrifolium* subsp. *barrelieri*, both accessions of *C. quadrifolium* subsp. *quadrifolium* and the accession of *C. quadrifolium* subsp. *parviflorum*. Ulig1 corresponds to *C. somedanum* (CENT63) and the accession CEN71 of *C. littorale* subsp. *uliginosum*, Port to *C. chloodes, C. somedanum* (CENT59) two accessions of *C*. *scilloides* and *C. portense*, Lit2 corresponds to two accessions of *C. littorale* subsp. *littorale* and *C. littorale* subsp. *uliginosum* (CENT56), Lin corresponds to two accessions of *C. quadrifolium* subsp. *linariifolium*. Ery2 corresponds to two accessions of *C. erythraea* subsp. *rhodense*, *C. turcicum*, two accessions of *C. erythraea* subsp. *erythraea* and *C. erythraea* var. *subcapitatum*, Maj corresponds to two accessions of *C. grandiflorum* subsp. *majus*, *C. erythraea* subsp. *rumelicum* and *C. suffruticosum*. Grand corresponds to two accessions of *C. grandiflorum* subsp. *grandiflorum*, Boi corresponds to both accessions of *C. grandiflorum* subsp. *boissieri* and Out corresponds to the outgroup (*Exaculum pusillum*, *Schenkia spicata* and *Blackstonia perfoliata*). Bootstrap support is displayed on the branches. (C) Genetic structure of the *Centaurium* individuals with *k* = 2. Each individual is represented by a vertical bar, with its label below. Genetic clusters are represented with different colours.

The Neighbor-Net of diploid, tetraploid and hexaploid *Centaurium* species (Fig. 3A) allows recognition of two clusters that coincide with the two main clades displayed in the phylogenetic reconstruction (Fig. 1). The depth of divergence between taxa is consistent with the branch lengths in the network (Fig. 3A). Short branches in the network indicate low divergence of species and parallel edges (representing alternative splits) indicate uncertainty potentially resulting from hybridization events that have occurred within *Centaurium* taxa.

### Identification of reticulation and hybridization events

The loglik scores obtained from the different SNaQ analyses based on different hmax values indicate that three hybridization events is the most probable scenario (Supplementary Data Fig. S2). One of the three hybridization events involved species from the two main clades whereas the other two occurred within the western clade (Fig. 3B). *Centaurium maritimum* (Mar), from the widespread clade hybridized with *C. malzacianum* (Mal) and currently 35.2 % of its genome (estimated from SNPs) comes from *C. malzacianum*. The ancestor of *C. grandiflorum* s.s. (Grand) and *C. grandiflorum* subsp. *boissieri* (Boi) hybridized with *C. quadrifolium* subsp. *linariifolium* (Lin), and currently the *C. grandiflorum* lineage has 49 % of *C. quadrifolium* subsp. *linariifolium*’s genome. The third identified hybridization event involves the clade formed by the two accessions of *C. quadrifolium* subsp. *barrelieri*, the one of *C. quadrifolium* subsp. *parviflorum* and both accessions of *C. quadrifolium* s.s. (Qua), which seems to have hybridized with *C. somedanum* or *C. littorale* subsp. *uliginosum* accession CENT71 (Ulig1), so that 24.3 % of the genome in taxa of the clade Qua comes from either of these two species (Fig. 3B).

Bayesian clustering analyses of *Centaurium* with fastSTRUCTURE revealed that the best number of genetic clusters to explain the structure of the dataset is two (Fig. 3C, Supplementary Data Fig. S3), which matches the western and widespread clades. These analyses identified four taxa showing admixed ancestry, with similar proportions of the two genetic groups: the tetraploid *C. serpentinicola* and the hexaploids *C. mairei*, *C. malzacianum* and *C. centaurioides.* One of the two accessions of the diploid *C. maritimum* (CENT29.2) also showed a percentage of admixture.

### *Allopolyploid origin of* Centaurium *species*

**Fig. 4.**
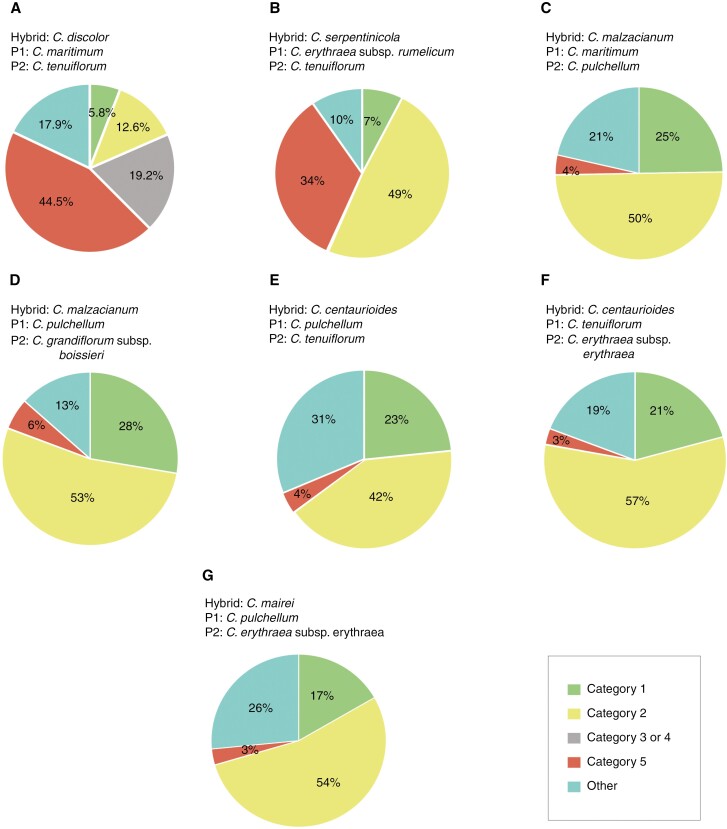
SNIPloid results for three *Centaurium* hybrid taxa (Hybrid) and its putative parentals (P1, parent 1; P2, parent 2). Categorization of SNPs of (A) *C. discolor*, (B) the hybrid *C. serpentinicola*, (C, D) *C. malzacianum*, (E, F) *C. centaurioides* and (G) *C. mairei*.

The allopolyploid origin of the tetraploid *C. discolor* proposed by Guggisberg *et al*. (2006), involving *C. maritimum* and *C. tenuiflorum*, was tested with SNIPloid (Fig. 4A). 44.5 % of the *C. discolor's* SNPs were classified in category 5 (the variations found in the genomes occur both in the hybrid and in one of the parental genomes), 19.2 % in category 3or4 (i.e. variations in SNPs occurring in *C. discolor* that could not be identified with certainty in either of the parent genomes), 17.9 % were classified as other (not falling into any of the defined categories), 12.6 % in category 2 and 5.8 % in category 1 (i.e. some alleles were specific to the genomes of *C. tenuiflorum* and *C. maritimum*, respectively).

Testing the hybrid ancestry of the tetraploid *C. serpentinicola* proposed by Mansion *et al.* (2005) using SNIPloid, 49 % of SNPs were found to be specific to *C. tenuiflorum* (category 2), 34 % fell in category 5, 10 % were classified as other and 7 % were specific to *C. erythraea* subsp. *rumelicum* (category 1) (Fig. 4B).

Testing the hybrid ancestry of the hexaploid *C. malzacianum* proposed by Mansion *et al*. (2005), which involves *C. maritimum* and *C. pulchellum*, 50 % of *C. malzacianum*’s SNPs were found in the genome of *C. pulchellum* (category 2), 25 % were specific to *C. maritimum* (category 1), 21 % were referred to category other and 4 % were classified in category 5 (Fig. 4C). When we considered our previous results and tested the ancestry of the same hexaploid with *C. pulchellum* and a species from the western clade (*C. grandiflorum* subsp. *boissieri)*, 53 % of the SNPs were specific to *C. grandiflorum* subsp. *boissieri* (category 2), 28 % were specific to *C. pulchellum* (category 1), 13 % fell in the category other and 6 % in category 5 (Fig. 4D).

Testing the hybrid ancestry of the hexaploid *C. centaurioides* according to Mansion *et al*. (2005), using *C. pulchellum* and *C. tenuiflorum* as parents, 42 % of the SNPs were also found in the genome of *C. tenuiflorum* (category 2), 23 % in the *C. pulchellum* genome (category 1), 31 % classified in the category other, and 4 % in category 5 (Fig. 4E). When we considered our previous results and tested the ancestry of this hexaploid species with *C. tenuiflorum* and a species from the western clade (*C. erythraea* subsp. *erythraea*) 57 % of *C. centaurioides* SNPs were specific to *C. erythraea* subsp. *erythraea* (category 2), 21 % were specific to C*. tenuiflorum* (category 1), 19 % were classified as other, and 3 % fell in category 5 (Fig. 4F).

Finally, testing the ancestry of *C. mairei* considering *C. pulchellum* and *C. erythraea* subsp. *erythraea*, 54 % were specific to *C. erythraea* subsp. *erythraea* (category 2), 17 % of *C. mairei* SNPs were specific to *C. pulchellum* (category 1), 26 % were classified as other, and 3 % fell in category 5 (Fig. 4G).

### Ploidy level and morphology

The stress score of the NMDS analysis for two dimensions was 0.0689427, indicating a low discrepancy between the original distances and those obtained after dimensionality reduction (Kruskal, 1964), as well as a correct fit of the graphical representation in the model.

**Fig. 5.**
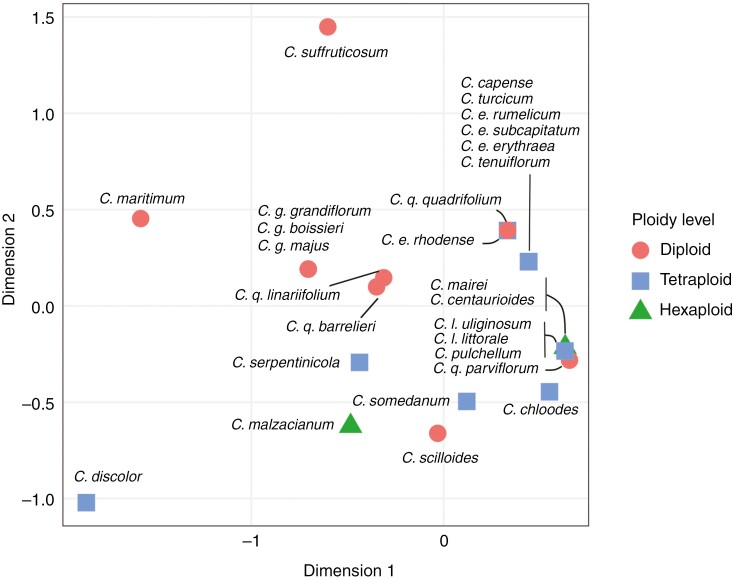
Two-dimensional NMDS showing the relationships between morphological traits and ploidy levels.

The NMDS ordination analysis revealed differences among ploidy levels for some morphological traits (Fig. 5). Specifically, based on the data provided in Supplementary Data Table S1, it can be inferred that most diploid *Centaurium* species had larger flowers and anthers than tetra- and hexaploid species. In addition, most diploid species showed no contact between stigmas and anthers, contrary to polyploid (both 4*x* and 6*x*) species, which showed low herkogamy. However, most species of the three ploidy levels showed zygomorphic androecium symmetry, curved style and short stigma (<0.7 mm). Regarding the flower display during anthesis, most diploid species had >30 flowers per plant, and hexaploids had <30 flowers per plant. Tetraploid species were mixed, with half having >30 and the other half <30 flowers per plant. Polyploid taxa (tetra- and hexaploids) and diploids were grouped separately considering their morphology, excluding *C. scilloides* and *C. quadrifolium* subsp. *parviflorum*, both diploids, which fell within the polyploid taxa. Three outliers were found: *C. maritimum*, *C. suffruticosum* and *C. discolor*.

## DISCUSSION

Applying phylogenomic approaches to reconstructing the phylogeny of *Centaurium* is a crucial step towards understanding its intricate evolutionary history, which has involved hybridization and polyploidy. Here we report the first phylogenetic reconstruction of the genus inferred using high-throughput sequencing (RADseq; Fig. 1) since previous phylogenetic studies were based on Sanger sequencing (Mansion and Struwe, 2004; Mansion *et al*., 2005; Jiménez-Lobato *et al*., 2019). Our study confirms the monophyly of the genus, as recognized in Mansion *et al*. (2005) and Jiménez-Lobato *et al*. (2019).

Consistent with previous phylogenetic reconstructions of the genus (Mansion and Struwe, 2004; Mansion *et al*., 2005; Jiménez-Lobato *et al.*, 2019, Maguilla *et al*., 2021), our tree includes two main clades, which Maguilla *et al*. (2021) termed the ‘western’ and ‘widespread’ clades. However, there are some incongruences with previous reconstructions based on nuclear regions (Mansion *et al*., 2005; Jiménez-Lobato *et al*., 2019). First, species such as *C. malzacianum*, *C. serpentinicola* and *C. tenuiflorum* fell into the opposite main clade compared with the nuclear reconstruction of Jiménez-Lobato *et al*. (2019) and in both reconstructions by Mansion *et al*. (2005), based on nuclear and plastidial sequences (Fig. 1). In addition, there are some differences within both the western and widespread clades. Specifically, our phylogenetic tree shows two subclades (i.e. A and B) within the western clade, whereas the reconstruction by Jiménez-Lobato *et al*. (2019) found no clear subclades. Also, the widespread clade in Jiménez-Lobato *et al*. (2019) included two subclades following the diversification of *C. malzacianum*, which were not confirmed in our tree.

The differences in topologies obtained from plastid and nuclear DNA regions compared with our RADseq reconstruction of polyploids may be due to several factors. The limited number of markers sequenced in previous studies compared with the number of regions sequenced here may be the main reason, as RADseq, unlike Sanger sequencing approaches, can recover genetic information from across the whole genome. Sanger sequencing of uniparentally inherited cpDNA regions used in previous studies only tracks the evolutionary history of one of the parents of a hybrid, not its full history (Rothfels, 2021). DNA regions, both nuclear and plastidial, have their own evolutionary histories (Kirschner *et al*., 2015; Fehrer *et al*., 2021; Rothfels, 2021), limiting their ability to serve as proxies for species level phylogenies, especially when the number of sequenced regions is small. In contrast, RADseq approaches retrieve genetic information from thousands of biparentally inherited coding and non-coding regions across the entire genome (Davey *et al*., 2011; McKain *et al*., 2018), which sheds light on the evolutionary history of challenging groups with polyploidy and hybridization.

### *Hybridization is a key event in the evolution of* Centaurium

Our study infers hybridization events (Figs 2–4; Supplementary Data Fig. S2) as we confirm an allopolyploid origin of four *Centaurium* species and document signatures of hybridization across the genus. This genus originated in the late Miocene in the Mediterranean Basin (Jiménez-Lobato *et al*., 2019). Two main factors have been reported to have fostered the diversification of the genus. During the Messinian salinity crisis (5.96–5.33 Mya), characterized by an extremely dry climate and land connections because of the evaporation of the seas (Duggen *et al.*, 2003), *Centaurium* could have exploited the novel environmental conditions due to its apparent resilience in dry conditions (Živković *et al*., 2007; Jiménez-Lobato *et al*., 2019). The onset of the Mediterranean climate (3.4–2.8 Mya) could have facilitated a new phase of diversification after a period of stasis (Jiménez-Lobato *et al*., 2019; Maguilla *et al*., 2021). These events could have favoured hybridization in the genus. The topology of our tree, consistent with the Neighbor-Net graph in relationships, relative distances among taxa, and recognizing two main clades, is partly congruent with the existing infrageneric taxonomy of the genus (subgenera and sections).

Four speciation processes occurred by hybridization events between the two main clades (i.e. the widespread and western clades) and also among subclades A and B in the western clade (Fig. 3B) (see below). The genetic structure estimated with Bayesian genetic clustering approaches is congruent with the occurrence of hybrid species and with the phylogeny. fastSTRUCTURE recognized two genetic clusters (Figs 1 and 3C), one corresponding to the western clade and the other to the widespread clade, including *C. discolor*, *C. tenuiflorum*, *C. pulchellum*, *C. capense* and *C. maritimum*. The allopolyploid condition of the hexaploid taxa *C. malzacianum*, *C. centaurioides* and *C. mairei* and the tetraploid *C. serpentinicola* is also reflected in fastSTRUCTURE since they appear admixed, containing similar proportions of the two genetic groups (Fig. 3C).

The allopolyploid origin of these four species has been confirmed by our data. However, the specific parentages hypothesized by Mansion *et al*. (2005) and Guggisberg *et al*. (2006) have only been partially confirmed. The parental species of the hexaploid *C. malzacianum* have been suggested to be *C. maritimum* and *C. pulchellum* (Mansion *et al*., 2005). However, based on the admixture pattern from our fastSTRUCTURE results (Fig. 3C), its parents could have been species from two main clades. We further tested the suggested allopolyploid origin with SNIPloid and concluded that the tetraploid *C. pulchellum* could be one of the parents (Fig. 4C). However, the role of *C. maritimum* as the other parent is more controversial, as only 25 % of the SNPs are specific to this species, compared with 21 % of SNPs in the hybrid corresponding to another species (Fig. 4C). In order to test the alternative hypothesis derived from fastSTRUCTURE, that the hybrid ancestry of *C. malzacianum* involves the two main clades, we performed SNIPloid with *C. pulchellum* from the widespread clade and *C. grandiflorum* subsp. *boissieri* from the western clade. Our results do not support the hypothesis of Mansion *et al*. (2005), as more than half of the SNPs correspond to a species from the western clade. The morphological resemblance of *C. malzacianum* to *C. maritimum*, on which Mansion *et al.* (2005) partly based their proposed parentage, could also be due to traces of ancient hybridization events, among other factors. This was also suggested in our analyses and is discussed below.

Regarding the hexaploid *C. centaurioides*, we confirm that *C. tenuiflorum* is one of its parents. However, 31 % of the SNPs do not belong to either of the two proposed parentals, but to another species which, based on our analyses (Fig. 3C), should come from the western clade. Our SNIPloid analysis with one randomly chosen species of the western clade (*C. erythraea* subsp. *erythraea*) instead of *C. pulchellum* confirmed the identification of the second parent from the western clade.

The hexaploid *C. mairei* was proposed to be an autopolyploid arising from tetraploid populations of *C. pulchellum* that produced both normal and unreduced gametes (Mansion *et al.*, 2005). This hypothesis was supported by the apparent lack of polymorphic sites in *C. mairei* and the weak divergence of sequences between accessions of *C. mairei* and *C. pulchellum* for *ITS* and *trnLF* regions (Mansion *et al*., 2005). However, our genomic results confirm the allopolyploid origin of *C. mairei*, rejecting its autopolyploid origin, but confirming the role of *C. pulchellum* as one of the parental species (Fig. 4G). Besides, the role of the western clade is also confirmed, as more than half of the SNPs in *C. mairei* correspond to a taxon randomly chosen from the western clade (*C. erythraea* subsp. *erythraea*). The tetraploid *C. serpentinicola* has been proposed to be an allotetraploid species whose parental species are *C. erythraea* subsp. *rumelicum* and *C. tenuiflorum*, based on their morphological similarities (Carlström, 1986; Zeltner, 1991). Mansion *et al*. (2005) could not find conclusive evidence for such a parentage in their molecular phylogenies. Our results shed light on this by confirming the hybrid origin of this tetraploid species and identifying one of the parental lineages, based on the finding that 49 % of *C. serpentinicola*’s SNPs match those in *C. tenuiflorum* (Fig. 4B). The finding that 34 % of *C. serpentinicola*’s SNPs are considered homoeo-SNPs, supports its allotetraploid origin.

We also studied the recent allopolyploid origin of the tetraploid *C. discolor* from *C. maritimum* and *C. tenuiflorum*, as previously suggested by Guggisberg *et al*. (2006) (Fig. 4A). However, we do not have consistent results. On one hand, previous studies (Guggisberg *et al*., 2006) and our SNIPloid estimations (Fig. 4A) do suggest this hybrid origin. Our SNIPloid results recover a high proportion of homoeo-SNPs (Fig. 4A), indicating that these SNPs are present in both the hybrid and parental genomes. On the other hand, our PhyloNetworks (Fig. 3B) and fastSTRUCTURE (Fig. 3C) results do not support that this taxon is a hybrid species. The hybridization events inferred by PhyloNetworks do not include this taxon, and the fastSTRUCTURE analysis infers only one genetic cluster within the species. However, it is important to note that this hybridization event may not have been detected because it is not an ancient event (Guggisberg *et al*., 2006).

We have also detected introgression in one of the samples of *C. maritimum* (CENT 29.2) (Fig. 3C), suggesting that this species hybridized with a congener from the western clade. In addition, as mentioned above, this diploid species has been considered to act as a parental species to *C. discolor* (Mansion *et al*., 2005; Guggisberg *et al*., 2006). This case involving *C. maritimum* suggests that this species might have been prone to hybridization.

The identification of parental taxa of hybrid *Centaurium* species has been conducted so far by cytogenetics, phylogenetic analyses based on Sanger sequencing and other molecular tools such as RAPD fingerprinting (Guggisberg *et al.*, 2006). However, the contribution of each parental genome to the hybrid genome cannot be adequately studied with these techniques. New innovative tools that use the results of high-throughput sequencing are helpful to identify parental taxa and to classify polymorphisms, in order to determine the genetic contribution of each parent (Peralta *et al*., 2013). Using these new methodological approaches, we may conclude that hybridization is ubiquitous across the genus *Centaurium*.

### Polyploidy, hybridization and phenotypic outcome

The ordination analysis of *Centaurium* taxa performed to explore the association between morphology and ploidy level (and to some extent, hybridization) revealed morphological differences between polyploids (tetraploids and hexaploids) and diploids (Fig. 5), which appears to be independent of their parental taxa and their geographical distribution. This relationship, where ploidy level variation promotes phenotypic changes, was first suggested by Stebbins (1950), and is confirmed in other genera, such as *Ranunculus* (Cires *et al*., 2010). One of the most common effects in the phenotypic outcome of polyploid plants is the gigas effect, which refers to the enlargement of plant traits (e.g. flowers, reproductive structures, leaves) in contrast to those of the diploids (Stebbins, 1971; Levin, 2002; Knight and Beaulieu, 2008). The mechanism underlying the gigas effect is associated with the relation between cell size and the amount of nuclear DNA, so that as the amount of DNA increases, so does the size of the cell (Segraves, 2017). However, exceptions to this effect have been documented, with polyploids exhibiting cell sizes similar to those in diploids (Clo and Kolář, 2021) or, in some cases, with polyploids displaying smaller traits than diploids (Vamosi *et al*., 2007; Ning *et al*., 2009). The genus *Centaurium* seems to be one of these exceptions, with diploids having larger flowers and anthers compared with those of polyploids (both tetra- and hexaploids), as well as more flowers per plant than polyploids.

Besides, regarding reproduction strategies in the genus, our analysis shows diploids displaying herkogamy, whereas polyploids display a higher physical proximity between anthers and style. However, no significant correlation between both floral size and ploidy level, and herkogamy and ploidy level, has been reported in the genus *Centaurium* (Jiménez-Lobato *et al.*, 2019). Then, the ordination analysis result supports a role of polyploidy in morphological diversification and thus, in speciation processes.

### Final remarks

It has long been recognized that hybridization and polyploidy are key processes in plant evolution (Abbott *et al*., 2013). The early stages of genome merging and doubling profoundly impact the molecular, genomic and physiological machinery, but they represent only a small fraction of the process compared with later evolutionary innovation (i.e. genome downsizing; Wang *et al*., 2021), which may remain latent until ecological opportunity dovetails with novel genomic/omic recombinants (Nieto Feliner *et al.*, 2020). In fact, biotic and abiotic stress responses in general are probably the most important and determining factors in the establishment and success of polyploids (Van de Peer *et al*., 2021). Key genome traits (such as chromosome number, genome size, repetitive DNA sequences, genes and regulatory sequences and their expression) evolve following polyploidy, generating diversity and possible novel traits, and enabling species diversification (Heslop-Harrison *et al*., 2023).

This study contributes the first phylogeny of the genus *Centaurium* performed with RADseq markers. This new phylogeny resolves the genealogical relationships among species, subspecies and lineages. The subsequent analyses demonstrate that this group of plants has undergone both ancient and recent hybridization events (many of them associated with polyploidy) that have resulted in a polyploid complex. The integrative approach used in this study, combining both phylogenetic and genomic analyses, sheds light on the study of polyploid and hybrid species and provides additional insights into their role as prominent forces in plant evolution. Specifically, this study in *Centaurium* constitutes an example of the importance of hybridization and polyploidization events during the Plio-Pleistocene in Mediterranean plants.

## SUPPLEMENTARY DATA

Supplementary data are available at *Annals of Botany* online and consist of the following. Table S1: binary classification of *Centaurium* characters used (from Jiménez-Lobato *et al*., 2019). Life-history traits were classified as annual/biennial (Ann) or perennial (Per). Figure S1: distribution of *Centaurium* taxa species used in the study. Each taxon is represented with a different colour (see legend). Figure S2: number of different hybridization events tested that have taken place in the genus. Figure S3: result of the chooseK tool, implemented in fastSTRUCTURE.

mcae066_suppl_Supplementary_Table_S1_Figures_S1_S3
