# Supplementary material for: Polyploidy and hybridization in the Mediterranean: unravelling the evolutionary history of Centaurium (Gentianaceae)
Source: Ann Bot. 2024 Apr 30;134(2):247–62. doi: 10.1093/aob/mcae066 (PMC11232519; doi:10.1093/aob/mcae066)
Supplement: mcae066_suppl_Supplementary_Table_S1_Figures_S1_S3 [file mcae066_suppl_supplementary_table_s1_figures_s1_s3.pdf]

Table S1. Binary classification of *Centaurium* characters used (from Jiménez-Lobato *et al*., 2019). Life-history traits were classified as annual/biennial (**Ann**) or perennial (**Per**). The flower display was the total number of flowers simultaneously present during anthesis and was coded as low (**Lo_fl**) (< 30 flowers per plant) or high (**Hi_fl**) (> 30 flowers per plant). The length of the flower (flower size) was coded as small-to-medium (**SM**) (< 15 mm) or large (**La**) (> 15.1 mm); the anther length as medium-to-large (**ML_anth**) (> 2.1 mm) or short (**Sh_anth**) (< 2 mm); the androecium symmetry was classified as radial (**Rad)** or zygomorphic (**Zig**); the style position as erect (**Er**) or curved (**Cur**); the herkogamy was classified as low (**Lo_herk**) (contact between stigmas and anthers) or high (**Hig_herk**) (with no contact between stigmas and anthers), and the stigma length was classified in medium-to-large (**ML_stig**) (> 0.8 mm) or short (**Sh_stig**) (< 0.7 mm).

**Taxa**	**ploidy**	**life cycle**	**flower display**	**flower size**	**anther length**	**androecium symmetry**	**style position**	**herkogamy**	**stigma length**
C. maritimum	diploid	Ann	Lo_fl	La	Sh_anth	Rad	Er	Hi_herk	ML_stig
C. discolor	tetraploid	Ann	Lo_fl	La	ML_anth	Rad	Er	Lo_herk	ML_stig
C. malzacianum	hexaploid	Ann	Lo_fl	La	ML_anth	Zig	Cur	Lo_herk	ML_stig
C. centaurioides	hexaploid	Ann	Lo_fl	SM	Sh_anth	Zig	Cur	Lo_herk	Sh_stig
C. pulchellum	tetraploid	Ann	Lo_fl	SM	Sh_anth	Zig	Cur	Lo_herk	Sh_stig
C. capense	tetraploid	Ann	Hi_fl	SM	Sh_anth	Zig	Cur	Lo_herk	Sh_stig
C. mairei	hexaploid	Ann	Lo_fl	SM	Sh_anth	Zig	Cur	Lo_herk	Sh_stig
C. tenuiflorum	diploid	Ann	Hi_fl	SM	Sh_anth	Zig	Cur	Lo_herk	Sh_stig
C. quadrifolium subsp. *quadrifolium*	diploid	Ann	Hi_fl	SM	ML_anth	Zig	Cur	Lo_herk	Sh_stig
C. quadrifolium subsp. *parviflorum*	diploid	Ann	Lo_fl	SM	Sh_anth	Zig	Cur	Lo_herk	Sh_stig

C. quadrifolium subsp. *barrelieri*	diploid	Ann	Hi_fl	La	ML_anth	Zig	Cur	Hi_herk	Sh_stig
C. quadrifolium subsp. *linariifolium*	diploid	Per	Hi_fl	La	ML_anth	Zig	Cur	Hi_herk	Sh_stig
C. serpentinicola	tetraploid	Ann	Lo_fl	La	ML_anth	Zig	Cur	Hi_herk	Sh_stig
C. chloodes	tetraploid	Per	Lo_fl	SM	Sh_anth	Zig	Cur	Lo_herk	Sh_stig
C. somedanum	tetraploid	Per	Lo_fl	La	Sh_anth	Zig	Cur	Lo_herk	Sh_stig
C. scilloides	diploid	Per	Lo_fl	La	ML_anth	Zig	Cur	Lo_herk	Sh_stig
C. littorale subsp. *littorale*	tetraploid	Ann	Lo_fl	SM	Sh_anth	Zig	Cur	Lo_herk	Sh_stig
C. littorale subsp. *uliginosum*	tetraploid	Ann	Lo_fl	SM	Sh_anth	Zig	Cur	Lo_herk	Sh_stig
C. erythraea subsp. *erythraea*	tetraploid	Ann	Hi_fl	SM	Sh_anth	Zig	Cur	Lo_herk	Sh_stig
C. erythraea var. *subcapitatum*	tetraploid	Ann	Hi_fl	SM	Sh_anth	Zig	Cur	Lo_herk	Sh_stig
C. erythraea subsp. *rhodense*	tetraploid	Ann	Hi_fl	SM	ML_anth	Zig	Cur	Lo_herk	Sh_stig
C. eryhtraea subsp. *rumelicum*	diploid	Ann	Hi_fl	SM	Sh_anth	Zig	Cur	Lo_herk	Sh_stig
C. turcicum	tetraploid	Ann	Hi_fl	SM	Sh_anth	Zig	Cur	Lo_herk	Sh_stig
C. suffruticosum	diploid	Ann	Hi_fl	SM	Sh_anth	Rad	Er	Hi_herk	ML_stig
C. grandiflorum subsp. *majus*	diploid	Ann	Hi_fl	La	ML_anth	Zig	Cur	Hi_herk	ML_stig
C. grandiflorum subsp. *grandiflorum*	diploid	Ann	Hi_fl	La	ML_anth	Zig	Cur	Hi_herk	ML_stig
. *grandiflorum* subsp. *boissieri*	diploid	Ann	Hi_fl	La	ML_anth	Zig	Cur	Hi_herk	ML_stig

Figure S1. Distribution of *Centaurium* taxa species used in the study. Each taxa is represented with different colors (see legend). Diploids are shown as circles, tetraploids as squares, and hexaploids as triangles.

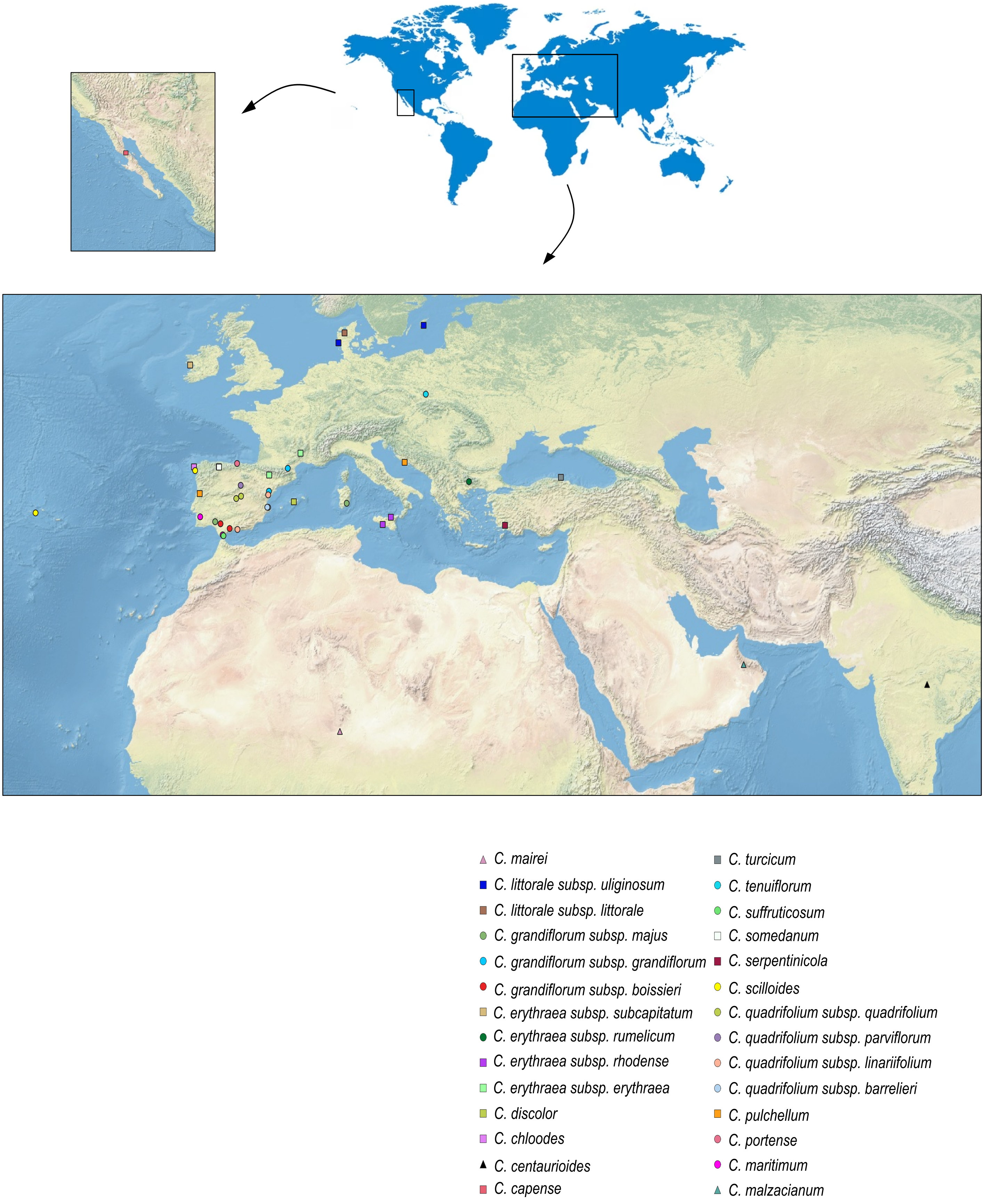

Figure S2. Number of different hybridization events tested that have taken place in the genus. X-axis indicates the number of hybridization events; the y-axis indicates the score of the pseudo-deviance of the network.

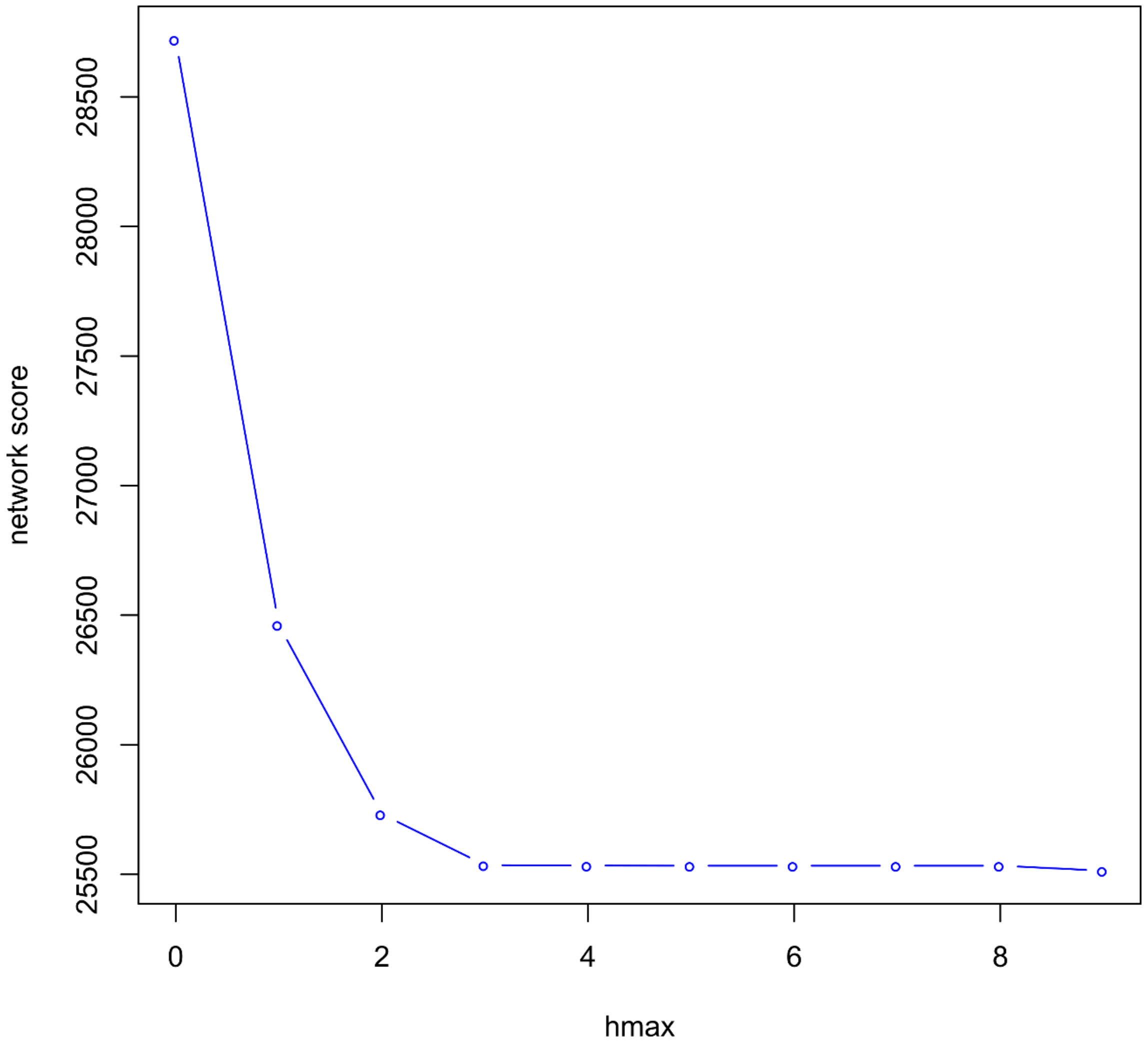

Figure S3. Result of the *chooseK* tool, implemented in fastStructure.

Model complexity that maximizes marginal likelihood = 2

Model components used to explain structure in data = 2
